# Influenza virus polymerase inhibitors in clinical development

**DOI:** 10.1097/QCO.0000000000000532

**Published:** 2019-02-28

**Authors:** Frederick G. Hayden, Nahoko Shindo

**Affiliations:** a Department of Medicine, University of Virginia School of Medicine, Charlottesville, Virginia, USA; b Health Emergencies Programme, World Health Organization, Geneva, Switzerland

**Keywords:** baloxavir, favipiravir, influenza, pimodivir, ribavirin

## Abstract

**Purpose of review:**

We review antivirals inhibiting subunits of the influenza polymerase complex that are advancing in clinical development.

**Recent findings:**

Favipiravir, pimodivir, and baloxavir are inhibitory in preclinical models for influenza A viruses, including pandemic threat viruses and those resistant to currently approved antivirals, and two (favipiravir and baloxavir) also inhibit influenza B viruses. All are orally administered, although the dosing regimens vary. The polymerase basic protein 1 transcriptase inhibitor favipiravir has shown inconsistent clinical effects in uncomplicated influenza, and is teratogenic effects in multiple species, contraindicating its use in pregnancy. The polymerase basic protein 2 cap-binding inhibitor pimodivir displays antiviral effects alone and in combination with oseltamivir in uncomplicated influenza, although variants with reduced susceptibility emerge frequently during monotherapy. Single doses of the polymerase acidic protein cap-dependent endonuclease inhibitor baloxavir are effective in alleviating symptoms and rapidly inhibiting viral replication in otherwise healthy and higher risk patients with acute influenza, although variants with reduced susceptibility emerge frequently during monotherapy. Combinations of newer polymerase inhibitors with neuraminidase inhibitors show synergy in preclinical models and are currently undergoing clinical testing in hospitalized patients.

**Summary:**

These new polymerase inhibitors promise to add to the clinical management options and overall control strategies for influenza virus infections.

## INTRODUCTION

Influenza causes serious health, economic, and societal impacts despite existing vaccines and antivirals. Currently, widespread resistance to adamantanes is present in circulating viruses, and neuraminidase inhibitors (NAIs) are the only effective antivirals available in most countries. However, global circulation of oseltamivir-resistant seasonal A(H1N1) virus occurred in 2008–2009 and NAI resistance remains a threat. Development and clinical application of new antivirals with different mechanism of action are therefore critically important. Recent progress in understanding the structure and functions of the influenza polymerase complex has facilitated the identification of several novel antivirals targeting individual components of the complex [1,2^▪^]. The polymerase heterotrimer is composed of three protein subunits that are highly conserved, interact closely, and are essential for efficient viral replication and associated virulence [3–6]. The polymerase basic protein 2 (PB2) subunit binds the 5’ cap (m7-GTP) of host pre-mRNAs and positions them for cleavage through the cap-dependent endonuclease located in the N-terminal domain of polymerase acidic protein (PA) subunit. This ‘cap-snatching’ process provides a RNA primer for transcription of viral mRNA by the RNA-dependent RNA polymerase function of polymerase basic protein1 (PB1). The transcriptase activity of this subunit is responsible for generating messenger, complementary, and virion RNAs.

This article provides a brief overview of the current development status of the most promising agents targeting the influenza virus polymerase complex (Table 1). There are many knowledge gaps for most of these agents, but all of them are inhibitory for influenza A viruses resistant to adamantanes and NAIs, so that the wider availability of one or more polymerase inhibitors would provide important therapeutic options. Furthermore, several of these agents show enhanced antiviral action when combined with NAIs and sometimes with one another in preclinical studies, so that combination therapy should increase antiviral potency and reduce the risk of antiviral resistance emergence. 

**Table 1:** Overview of polymerase inhibitors approved or in advanced clinical development

Feature	Favipiravir^a^ (T-705)	Pimodivir (JNJ-63623872)	Baloxavir^b^ (S-033188)
Influenza polymerase target	PB1	PB2	PA
Influenza virus-type spectrum	A, B, C	A	A, B
Inhibition of M2I and NAI-resistant viruses	Yes	Yes	Yes
In-vitro potency	μM	nM	nM
Synergy with NAIs for influenza A viruses	Yes	Yes	Yes
Route of dosing	Oral (intravenous under development)	Oral (intravenous under development)	Oral
Antiviral efficacy in uncomplicated influenza	Yes	Yes	Yes
Clinical efficacy in uncomplicated influenza	Variable	Not formally tested	Yes
Emergence of variants with decreased in-vitro susceptibility during monotherapy	Not to date	Yes, common	Yes, common

PA, polymerase acidic protein; PB, polymerase basic protein; NAI, neuraminidase inhibitor. M2I, M2 ion channel inhibitor.

^a^Approved for novel strains unresponsive to current antivirals in Japan in 2014 (trade name, Avigan).

^b^Approved for influenza treatment in 2018 in Japan and United States (trade name, Xofluza).

**Box 1:**
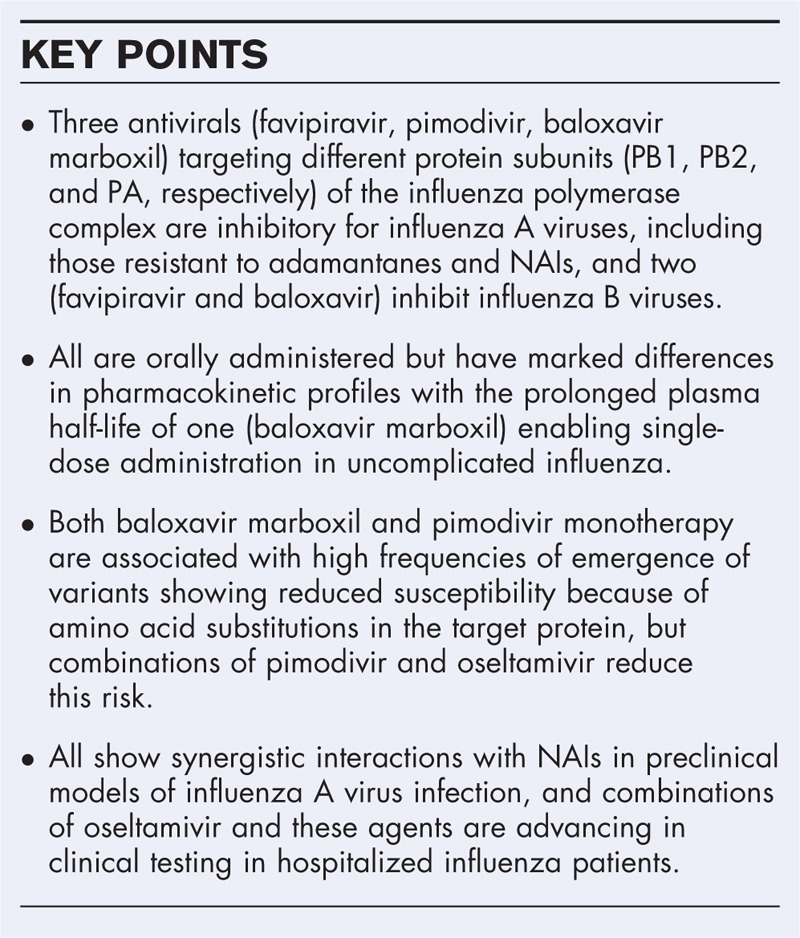
no caption available

## RIBAVIRIN

The older PB1 transcriptase inhibitor ribavirin has been administered orally, by aerosol, or intravenously in past influenza studies, but these have not shown convincing clinical efficacy [7]. One recent double-blinded randomized, controlled trial (RCT) tested a combination (termed Triple Combination Antiviral Drug or TCAD) of oral amantadine, ribavirin, and oseltamivir that had shown greater effectiveness than single agents or dual combinations in preclinical models including those employing viruses resistant to amantadine. Outpatients at higher risk for influenza complications who presented within 5 days of symptom onset were randomized to TCAD (oral oseltamivir 75 mg, amantadine 100 mg, and ribavirin 600 mg) twice daily (BID) or oseltamivir [8^▪▪^]. Among the 394 with proven influenza virus infection, TCAD was associated with significantly greater antiviral effects than oseltamivir monotherapy (40.0% of TCAD versus 50.0% of oseltamivir recipients had detectable viral RNA on day 3) but somewhat less rapid resolution of several illness measures, probably related to the side-effects of the TCAD regimen [8^▪▪^]. More serious adverse events and hospitalizations occurred in the TCAD group. Thus, this triple drug regimen failed to improve clinical outcomes compared to oseltamivir alone in an outpatient cohort at increased risk for influenza complications.

## FAVIPIRAVIR (T-705)

Favipiravir is a substituted pyrazine derivative that inhibits the replication of many RNA viruses including influenza A, B, and C viruses. It was approved in Japan in 2014 with an indication limited to treatment of novel or reemerging influenza virus infections unresponsive or insufficiently responsive to current agents but remains investigational in other countries.

## PRECLINICAL STUDIES

Once ribosylated and phosphorylated intracellularly, favipiravir triphosphate acts as a purine nucleoside analogue that functions as a competitive substrate inhibitor of the viral RNA-dependent RNA polymerase [9^▪^]. A key mechanism of antiviral action is thought to be lethal mutagenesis related to an increased guanosine to adenine mutation frequency causing nonviable progeny during replication [10].

Favipiravir inhibits replication of seasonal influenza A and B viruses, including those resistant to adamantanes and NAIs, and avian A(H5N1), A(H7N9), viruses, at 50% effective inhibitory concentrations (EC_50_s) of 0.03–0.94 μg/ml, 0.09–0.83 μg/ml, and 0.06–3.53 μg/ml, respectively [9^▪^]. Favipiravir shows dose-dependent reductions in mortality and lung viral titers in murine models of influenza including infections by A(H5N1) and A(H7N9) viruses [11,12] with enhanced antiviral efficacy when combined with NAIs [9^▪^]. Combined treatment with oseltamivir and favipiravir resulted in 100% survival in mice infected with an A(H5N1) virus and extended the treatment window to 96 h postinfection [13]. In a highly immunocompromised nude mouse model, prolonged therapy with favipiravir was more effective than NAIs in extending survival, but combination therapy with an NAI variably reduced lung viral titers and did not prevent the emergence of NAI-resistant variants [14].

Most studies have found either no or only modest reductions in susceptibility during in-vitro passage in the presence of favipiravir [10,15,16]. Recently, a A(H1N1)pdm09 variant with 30-fold reduced susceptibility and substitutions in PB1 and polymerase acidic protein has been generated [17^▪^].

Favipiravir is associated with reversible histopathological changes in the testis and abnormal sperm in animals. Early embryonic deaths in rats and teratogenicity in multiple species have been observed with exposure levels similar to or lower than those in humans [18]. In juvenile animals, favipiravir causes abnormalities in musculature and mortality during prolonged dosing.

### Clinical studies

Favipiravir has complex, nonlinear, time and dose-dependent pharmacokinetics that are affected by weight [18,19]. Because favipiravir is both metabolized by and inhibits aldehyde oxidase, initial oral loading is required to obtain adequate blood levels. Although the plasma T_1/2elim_ of favipiravir is approximately 4 h (Table 2), human data on intracellular concentrations of the triphosphate in the respiratory tract are lacking. Furthermore, the lower than predicted blood levels observed in Ebola virus disease and severe influenza patients raise concerns about bioavailability and/or altered metabolism in seriously ill persons [20,21]. Favipiravir or its metabolites have been detected in semen and breast milk.

**Table 2:** Summary of human pharmacokinetic features for oral influenza polymerase inhibitors approved or in advanced clinical development

Feature	Favipiravir	Pimodivir	Baloxavir marboxil
Usual dose regimen in adults with uncomplicated influenza^a^	1800 mg BID on day 1 followed by 800 mg BID on days 2–4^a^	600 mg BID for 5 days	Single dose of 40 mg for weight <80 kg, 80 mg for weight ≥80 kg
Oral bioavailability (estimated)	High (>95%)	∼46% (tablet)	High. Baloxavir marboxil is rapidly hydrolyzed by esterases in the small intestine, blood, and the liver
Time to maximum concentration (C_max_)	0.5–3.0 h	0.5–6 h	1.5–3.5 h
Plasma concentrations in adults	C_max_ of ∼35–50 μg/ml from Day 2^a^	Mean C_max_ and C_min_ of 1,590 ng/ml and 345 ng/ml, respectively, at steady state	Mean (CV%) C_max_ of baloxavir acid 96.4 ng/ml (45.9%) and at 40 mg dose (weight<80 kg) and 107 ng/ml (47.2%) at 80 mg dose (weight ≥80 kg)
Effect of food	No important effect	No effect on AUC but ∼50% higher C_max_	Decrease in C_max_ and AUC_0-inf_ of baloxavir acid by ∼48% and ∼38%, respectively
Plasma protein binding	54%	99%	93%
			
Plasma elimination half-life (T1/2elim)	∼2–5.5 h	13–28 h	49–91 h (baloxavir acid)
Primary route of elimination	Renal clearance of metabolites	Feces (∼95%)	∼80% feces and 18% in urine
Metabolism	Hydroxylation by aldehyde oxidase and xanthine oxidase. Glucuronidated metabolite also found	Metabolized (<10% in humans) by cytochrome P450 (CYP) 3A and aldehyde oxidase followed by glucuronidation	Baloxavir acid is primarily metabolized by uridine diphosphate glucuronosyl transferase 1A3 with minor contribution from CYP3A4
Dose reductions in renal insufficiency	No reductions currently recommended by manufacturer	ND	No, but ND for severe impairment
Dose reductions in hepatic impairment	Yes, with with severe hepatic impairment (Child-Pugh class C)	ND	ND for severe impairment. No need with moderate hepatic impairment (Child–Pugh class B)
Cytochrome P450 and other interactions	Inhibits aldehyde oxidase and CYP2C8, but does not induce CYP enzymes	Substrate of P-glycoprotein and both a substrate and inhibitor of the organic anion transporting polypeptide 1B1	Baloxavir marboxil inhibits CYP2B6, CYP2C8, and CYP3A4 activities, and baloxavir acid CYP2B6 and CYP3A4 activities but no clinically relevant interactions currently recognized
Potential drug interactions of concern	Acetaminophen – dosing of acetaminophen should be no more than 3000 mg/day (or less in patients with hepatic insufficiency). Theophylline coadministration increases favipiravir C_max_ and AUC by about 30%. Other precautions include co-administration with pyrazinamide, repaglinide, and famciclovir	Not reported to date	Coadministration with dairy products, calcium-fortified beverages, polyvalent cation-containing laxatives, antacids or oral supplements (e.g., calcium, iron, magnesium, selenium, or zinc) may decrease plasma levels and should be avoided

AUC, area under the concentration-time curve; CV, coefficient of variation; ND, not determined.

^a^Dose regimen tested in phase 3 RCTs in uncomplicated influenza. Approved favipiravir dose regimen in Japan is 1,600 mg BID on day 1, followed by 600 mg BID on days 2–5. Estimated concentrations are based on doses of 800 mg BID on days 2–5 in healthy subjects. Adapted from [18,19,21,30,38,39].

Favipiravir does not show pharmacokinetic interactions with oseltamivir [18,19]. Higher plasma maximum concentration (C_max_) and area under the concentration–time curve (AUC) levels of favipiravir occur in patients with increasing degrees of hepatic function impairment, but guidelines for dose adjustment are not currently available. Favipiravir has multiple potential drug–drug interactions (Table 2) [18]. In healthy volunteers co-administration of acetaminophen and favipiravir significantly increased (by about 20%) overall exposure to acetaminophen (Table 2) [22].

Multiple unpublished clinical studies with varying dose regimens have been conducted in adults with acute, uncomplicated influenza. One dose-ranging RCT in uncomplicated influenza (NCT01728753) found that a BID dosing regimen (1800 mg BID on day 1 and 800 mg BID on days 2–5) gave better antiviral and clinical effects than a three times daily (TID) dosing regimen. The favipiravir 1800 mg/800 mg BID group also demonstrated significantly faster time to alleviation of influenza symptoms (median, 82.3 versus 97.3 h) and viral load reductions compared with the placebo group [23]. Two international placebo-controlled, phase 3 RCTs of favipiravir have been completed in adults with uncomplicated influenza (NCT02008344; NCT02026349). These RCTs tested a favipiravir regimen that consisted of two 1800 mg loading doses on day 1 followed by 800 mg BID on days 2–5. Among influenza-infected study participants, one study (*N* = 594) found a significant difference of 14.2 h in median time to alleviation of symptoms (TTAS) and faster reductions in nasal virus titers in the favipiravir recipients compared with placebo, but the other study (*N* = 668) found only a 6.1 h difference in time to illness alleviation [18,24]. No studies in seriously ill or hospitalized influenza patients or in children have been reported to date. However, an open-label, dose-escalating study of favipiravir pharmacokinetics has been initiated in hospitalized influenza patients treated with oseltamivir in China (NCT03394209). Further studies at higher doses and in combination with NAIs are needed to determine its safety and efficacy in high-risk and seriously ill influenza patients.

The in-vitro favipiravir susceptibility of A(H1N1)pdm09 and A(H3N2) viruses isolated before or 1–2 days after favipiravir treatment showed no significant changes [25^▪^]. However, some posttreatment isolates had amino acid substitutions in PB1, PB2, and/or PA, the significance of which remains to be determined.

Warnings in the Avigan labeling [18] include that favipiravir is contraindicated in women who might be or are pregnant and in lactating women because of its association with embryonic deaths and teratogenicity in animal studies (above) and that men should use the most effective contraceptive methods including condoms in sexual intercourse and not to have sexual intercourse with pregnant women during treatment and for 7 days afterwards. Favipiravir has been reasonably well tolerated in clinical studies, although it is associated with dose-related, asymptomatic increases in serum uric acid levels and should be used with care in patients with gout or a history of gout and in those with hyperuricemia. Other adverse events may include mild to moderate diarrhea, asymptomatic increase of transaminases, and uncommonly decreased neutrophil counts [19].

## PIMODIVIR (JNJ-63623872, FORMERLY VX-787)

Pimodivir is a novel, cyclohexyl carboxylic acid analogue inhibitor of the PB2 cap-binding subunit of influenza A viruses that has advanced to phase 3 testing in hospitalized patients and high-risk outpatients with influenza A virus infections (Table 3).

**Table 3:** Randomized controlled phase 3 treatment efficacy trials of influenza polymerase inhibitors in progress

Drug (Study number)	Target population	Enrollment criteria	Intervention	Target enrollment	Primary outcome measure	Comment
Pimodivir (NCT03376321)	Hospitalized, ages 13–85 years	lllness duration ≤96 h, RT-PCR positivity for influenza A virus, baseline NEWS ≥4, and peripheral capillary oxygen saturation <94% on room air	Pimodivir 600 mg BID for 5 days (with investigator discretion to extend therapy to 10 days) and SOC (may include influenza antivirals or supportive care only) versus SOC and placebo	600	Day 6 clinical status assessed by Hospital Recovery Scale: not hospitalized; non-ICU hospitalization, not requiring supplemental oxygen; non-ICU hospitalization, requiring supplemental oxygen; admitted to the ICU, not requiring invasive mechanical ventilation; requiring invasive mechanical ventilation; and death	Initiated fall 2017. Severe hepatic insufficiency or concurrent antiviral therapy for chronic HCV, recent MI, unstable angina pectoris, significant atrial or ventricular arrhythmias are exclusion criteria
Pimodivir (NCT03381196)	Adolescent, adult, and elderly outpatients (ages 13–85 years) at risk of developing complications	RT-PCR or RIDT positivity for influenza A, at least 1 respiratory symptom and at least 1 systemic symptom of moderate or greater severity, and illness duration ≤72 h, study participants 13–65 years of age must also have at least 1 risk factor for influenza complications (not including pregnancy)	Pimodivir 600 mg BID for 5 days (with investigator discretion to extend therapy to 10 days) and SOC (may include influenza antivirals or supportive care only) versus SOC and placebo	720	Time to resolution of influenza-related symptoms as assessed by the patients using Flu Intensity and Impact Questionnaire (FluiiQ)	Initiated fall 2017. Severe immune-compromise, severe hepatic insufficiency or concurrent antiviral therapy for chronic HCV, recent MI, unstable angina pectoris, significant atrial or ventricular arrhythmias are exclusion criteria
Baloxavir marboxil (NCT02949011)	Adult and adolescent (age ≥12 years) outpatients with underlying condition associated with increased risk of influenza complications	Fever ≥38°C (axillary), positive RIDT result or a patient with a negative RIDT may be enrolled contact with a known case within the prior 7 days and at least 1 systemic and 1 respiratory symptom of moderate or greater severity, and duration of illness ≤48 h	Baloxavir 40 or 80 mg once (depending on weight) and placebo for oseltamivir versus placebo for baloxavir and oseltamivir 75 mg BID for 5 days versus matching placebos for both	2157 (actual)	Time to improvement of influenza symptoms, defined as the time from initiation of study drugs to improvement of influenza symptoms for at least 24 h	Trial initiated 2016; enrollment completed 2018. Women who were pregnant or breastfeeding, those requiring systemic antibiotic therapy, those with hepatic impairment, known creatinine clearance ≤60 ml/min, or multiple types of serious immune-suppression were excluded
Baloxavir marboxil (NCT03684044)	Hospitalized adolescents, adults aged ≥12 years	Illness duration ≤96 h; influenza A or B confirmed by RIDT or RT-PCR, NEWS2 score of ≥4, and need for ventilatory or supplemental oxygen support or influenza-related complication influenza (e.g., pneumonia) requiring hospital care	Baloxavir marboxil (weight-based dose) on days 1 and 4 with a third dose on day 7 for those not improved by day 5 versus placebo (2:1 ratio). Both groups receive SOC NAI per local practice	240	Time to clinical improvement defined as: Time to hospital discharge or time to NEWS2 of ≤2 maintained for 24 h up to day 35	Initiated November 2018; estimated completion July 2021. Exclusions include pregnancy, breastfeeding, weight <40 kg, known severe renal impairment (estimated glomerular filtration rate <30 ml/min/1.73 m^2^) or dialysis, severe liver function abnormalities
Baloxavir marboxil (NCT03629184)	Otherwise healthy children 1 to <12 years old	Influenza-like illness including fever ≥38°C and at least 1 respiratory symptom ≤48 h duration	Baloxavir administered as oral suspension in a single dose on day 1 and oseltamivir placebo versus oseltamivir administered as oral suspension BID for 5 days and baloxavir placebo	120	Percentage of participants with adverse events and serious adverse events up to day 29	Initiated December 2018; estimated completion June 2020. Excludes risk groups and those requiring inpatient management. Weight-based dosing of baloxavir and oseltamivir (nonpaid consulting disclosures: Cidara and Gilead)

SOC, standard of care; NEWS, National Early Warning Score; HCV, hepatitis C virus; MI, myocardial infarction; RIDT, rapid influenza diagnostic test. Adapted from clinicaltrials.gov.

### Preclinical studies

Pimodivir targets the PB2 subunit of influenza A virus polymerase complex [26]. Single-cycle studies in cell culture indicate that delayed pimodivir addition at 6 h rapidly stops viral mRNA production and prevents cell death, unlike NAIs tested under the same conditions.

Pimodivir inhibits a wide range of influenza A viruses including NAI and adamantane-resistant isolates [26,27^▪^], but has little or no activity for influenza B viruses. Depending on the strain and assay method, EC_50_ values range from 0.13 to 22.9 nM in cell culture. Low pimodivir doses also provide enhanced survival compared with oseltamivir in murine models [28]. In a lethal murine model of A/Vietnam/1203/2004(H5N1) virus infection, pimodivir treatment showed dose-related reductions in mortality up to 120 h after viral challenge, whereas oseltamivir had little mortality benefit started at 24 h [27^▪^]. In-vitro pimodivir demonstrates synergy with oseltamivir, zanamivir, and favipiravir [27^▪^].

In-vitro passage of influenza A virus in presence of pimodivir selects for substitutions in PB2. One variant detected in experimentally infected volunteers (M431I in PB2) shows reduced replication efficiency in vitro [29]. The possible effects of other substitutions on viral fitness have not been reported.

No data are currently in the public domain regarding preclinical safety or maternal–fetal and juvenile toxicology.

### Clinical studies

Formulations in development include tablets for oral administration and a solution for intravenous administration (NCT02659735). Oral pimodivir demonstrates dose proportional pharmacokinetics (Table 2) with a mean terminal plasma T_1/2elim_ of about 24 h [24,30], such that steady-state levels are reached between days 3–4 of dosing [31^▪▪^]. It is metabolized by CYP 3A4, but has no effect on cytochrome P450 activity. The low renal elimination (∼5%) suggests that pimodivir does not require dose adjustments for renal insufficiency. No meaningful pharmacokinetic differences were found in hospitalized influenza patients between younger (aged 18–64 years) and elderly (65–85 years) adults [32]. In a drug–drug interaction study, oseltamivir increased pimodivir C_max_ by 31% with no change in C_min_ or AUC_12 h_; pimodivir had no effect on oseltamivir [24,30].

In experimentally infected volunteers, pimodivir regimens (100 mg daily, 400 mg daily, 900 mg once followed by 600 mg daily, or 1200 mg once followed by 600 mg daily) for total of 5 days were associated with reductions in nasal infectious virus titers compared with placebo (median AUC, 1.3, 0.7, 3.2, 0.4, and 5.9 log_10_ TCID_50_/ml∗day, respectively). The highest dose group also experienced significantly lower and more rapid resolution of influenza-like symptoms compared with placebo [29]. In a phase 2b dose-ranging study in uncomplicated seasonal influenza, pimodivir 600 mg BID for 5 days resulted in a significantly greater decrease in nasal viral RNA loads (AUC from day 1–8) compared with placebo and a somewhat greater reduction than the 300 mg BID dose regimen [31^▪▪^]. Pimodivir 600 mg combined with oseltamivir 75 mg BID resulted in a significantly lower viral RNA load AUC compared with pimodivir 600 mg alone. Both treatment groups appeared to have reduced risk of complications and nonsignificant acceleration of symptom resolution. A phase 2b trial (NCT02532283) comparing pimodivir 600 mg BID and oseltamivir to oseltamivir monotherapy in hospitalized adults found no significant overall differences in recovery, but a lower incidence of influenza-related complications was observed with the combination (5/63, 8%) compared with oseltamivir alone (5/32, 16%) [32]. In the subgroup enrolled within 72 h of symptom onset, the combination showed trends toward shorter durations of virus detection and illness. Pivotal phase 3 placebo-controlled RCTs of pimodivir combined with standard of care (SOC) (expected to include an NAI) in hospitalized adolescents and adults (NCT03376321) and in higher risk outpatients (NCT03381196) were initiated in the 2017–2018 influenza season (Table 3).

PB2 substitutions conferring reduced pimodivir susceptibility were observed in ∼10% experimentally infected, pimodivir-treated volunteers [29]. These were noted in the subset of study participants with high viral titers and high drug levels, which suggest that the substitutions resulted in a lack of inhibition in vivo. The PB2 M431I variant, which confers a 57-fold reduction in pimodivir susceptibility in cell culture, was the most common but other substitutions occurred. A similar overall frequency (10 of 58 study participants with available data) of detecting PB2 substitutions (S324K/N/R, F325L, S337P, K376N/R, T378S, and N510K) was found with pimodivir monotherapy in outpatients with uncomplicated influenza, but the combination with oseltamivir greatly reduced this risk (1 of 17 study participants) [31^▪▪^]. In the hospital-based study, no PB2 substitutions at relevant positions were observed in the combined pimodivir and oseltamivir group [32].

Among those administered a single dose up to 3200 mg, and patients given multiple doses of 600 mg, the most common adverse event has been dose-related diarrhea, usually characterized as ‘loose stools’ and mild in severity [24]. The self-limited diarrhea has occurred in 27% of outpatients dosed at 600 mg BID. The mechanism remains to be determined. Other adverse events possibly related to pimodivir include nausea, emesis, elevations in transaminases, and decreased neutrophil counts [31^▪▪^].

## BALOXAVIR MARBOXIL (S-033188)

Baloxavir marboxil is an oral prodrug that is rapidly converted to its active form baloxavir acid (formerly S-033447), a potent inhibitor of influenza cap-dependent endonuclease function. Baloxavir (Xofluza^TM^, Genentech USA, Inc., South San Francisco, CA; Shionogi & Co., Ltd., Osaka, Japan) was approved for treatment of uncomplicated influenza A and B virus infections in 2018 in Japan (for those weighing ≥10 kg) and the United States (for those aged 12 years and older).

### Preclinical studies

Baloxavir acid selectively inhibits the cap-dependent endonuclease activity of the polymerase acidic protein subunit required for transcription of influenza A and B viruses through binding with divalent cations in the active enzyme site [33,34^▪^]. In cell culture, baloxavir acid inhibits replication of representative influenza A viruses, including strains resistant to NAIs and adamantanes, at ∼0.5–1.6 nM and influenza B viruses at 2.2–6.5 nM concentrations [33]. The median EC50 values for clinical isolates range from 1.3 to 1.6 nM (0.63–0.77 ng/ml) for A(H1N1)pdm09, 0.74–1.4 nM (0.36–0.68 ng/ml) for A(H3N2), and 5.6–8.5 nM (2.7–4.1 ng/ml) for type B viruses [33,34^▪^]. Baloxavir shows greater antiviral and clinical effects in murine models of influenza A and B virus infection than oseltamivir [35], and in a murine model of A(H7N9), baloxavir showed dose-related antiviral effects and protection against mortality to a greater extent than oseltamivir [36]. Baloxavir acid demonstrates synergy in vitro with NAIs, and in mice, suboptimal baloxavir marboxil and oseltamivir combinations show enhanced antiviral efficacy [35].

Testing of passaged laboratory strains or clinical isolates from treated patients has identified isoleucine-to-threonine, methionine, and phenylalanine substitutions at amino acid position 38 in the polymerase acidic protein (PA/I38T/F/M) that confer at least 10-fold reduced susceptibility to baloxavir acid in influenza A viruses [33,34^▪^]; for influenza A(H3N2) and A(H1N1) the PA/I38T substitution confers approximately 60 and 30-fold reductions in susceptibility, respectively. Influenza a viruses harboring the PA/I38T substitution show reduced endonuclease activity and impaired replicative fitness in cell culture [34^▪^]. Another study found that the PA/I38T substitution was stably maintained during cell culture passage without drug pressure and that variants with only the PA/I38T substitution maintained high levels of replication [37]. Replication and transmission fitness studies with PA/I38X variants are in progress in animal models.

Monkeys showed elevations in liver function tests, including alanine aminotransferase, at doses of 20 mg/kg/day or higher. Baloxavir has not shown maternal or juvenile toxicity or adverse effects on reproduction or embryo-fetal development in animal models at exposure levels that exceed those observed with the maximally recommended human dose [38]. No effects on the central nervous, cardiovascular, and respiratory systems have been detected in preclinical studies to date at exposure levels well above those anticipated in humans.

### Clinical studies

Baloxavir marboxil shows nearly dose-proportional pharmacokinetics [38,39]. The prodrug is quickly hydrolyzed to baloxavir acid which has a prolonged plasma terminal T_1/2elim_ enabling single dose administration in uncomplicated influenza (Table 2). Administration with or shortly after food reduces exposure (Table 2), and absorption in seriously ill patients remains to be determined. Weight-based dosing is required to achieve similar drug exposures. The C_max_ after a 40 and 80 mg dose averaged 123 and 253 ng/ml, respectively, in Japanese study participants weighing 50–80 kg [39], but ∼35% lower levels are found in non-Asian study participants. Baloxavir does not show relevant pharmacokinetic interactions with oseltamivir [40].

A phase 2 RCT in 400 Japanese adults with uncomplicated influenza testing single oral doses of baloxavir (10, 20, or 40 mg) determined that the median TTAS was reduced by 23.4 to 28.2 h in baloxavir groups compared with placebo [41^▪▪^]. Significantly greater reductions in nasal viral loads compared to placebo were seen by one day after dosing. A phase 3 placebo- and oseltamivir-controlled RCT tested single baloxavir doses (40 mg for weight 40 to <80 kg; 80 mg for weight ≥80 kg) in 1064 uncomplicated influenza patients aged 12–64 years, 85–88% of whom had influenza A(H3N2) infection [41^▪▪^]. The median TTAS was 53.7 h in baloxavir recipients compared with 80.2 h in placebo (*P* < 0.0001) and to 53.8 h in oseltamivir recipients. Baloxavir was associated with significantly greater reductions in infectious virus and viral RNA titers than placebo or oseltamivir by one day after dosing. A phase 3 RCT of single-dose baloxavir treatment in higher-risk outpatients completed in the 2017–2018 season (Table 3) found that baloxavir significantly shortened the time to improvement of influenza symptoms by about one day, reduced complications and antibiotic use, and reduced viral titers compared with placebo [42]. Oseltamivir had similar effects in A(H3N2) infections, but baloxavir was associated with significantly greater antiviral and clinical effects in influenza B virus infections compared with oseltamivir. An open-label pediatric trial in Japan reported clinical and virologic effects in otherwise healthy children aged 6 months-12 years [43]. Further trials in children, including infants from birth (NCT03653364), are ongoing (Table 3). A RCT of baloxavir's efficacy and safety, administered in multiple doses in combination with a SOC NAI, in hospitalized influenza patients was launched in the 2018–2019 season (Table 3).

Emergence of PA/I38T/F/M variants conferring reduced susceptibility occurred in 2.3% and 9.7% of baloxavir recipients in the phase 2 [all A(H1N1)] and phase 3 [all A(H3N2)] RCTs, respectively [41^▪▪^]. Baloxavir recipients developing such variants showed transient rebounds in virus titers, prolonged virus positivity, and compared with recipients without variants, early delay in illness resolution. In a pediatric study, 23% of baloxavir-treated children had PA/I38X variants detected at day 6 or 9 [43]. The possible transmissibility of such variants requires careful study and monitoring, and household-based trials examining baloxavir's effects on transmission of wild-type and variant viruses are planned. Other non-I38X polymerase acidic protein substitutions were also noted in the clinical trials, but their significance remains to be determined.

No adverse events specifically related to single-dose baloxavir treatment have been identified to date. In the two RCTs, adverse events were reported in 21–27% of baloxavir, 25–29% of placebo, and 25% of oseltamivir recipients. Adverse events associated with cessation of study drug occurred in 0.3–0.4% across groups [41^▪▪^].

## CONCLUSION

Three antivirals that target different protein subunits of the influenza polymerase complex are in advanced clinical development, with one (baloxavir) already approved in both the United States and Japan. All are inhibitory for influenza A viruses resistant to adamantanes and NAIs, show synergistic interactions with NAIs in preclinical models, and are orally administered. Favipiravir's human pharmacokinetics are complex, requiring high loading doses, and its teratogenic effects in multiple species contraindicate its use in pregnancy. Studies in uncomplicated influenza have shown adequate tolerability but inconsistent clinical benefits, and the possible value of higher dose regimens in serious influenza remains to be determined. The influenza A-specific PB2 inhibitor pimodivir has shown significant antiviral activity in initial clinical studies and has been generally well tolerated except for diarrhea. Variants with reduced susceptibility emerge readily during pimodivir monotherapy, but combinations of pimodivir and oseltamivir show enhanced antiviral activity and reduce the frequency of emergence of such variants in both outpatients and those hospitalized with influenza; two placebo-controlled, phase 3 RCTs, one comparing the combination of pimodivir and SOC (predicted to be largely oseltamivir) to SOC in hospitalized influenza patients and the other in high-risk outpatients, are in progress. Baloxavir is a well-tolerated inhibitor of the PA cap-dependent endonuclease with a favorable human pharmacologic profile enabling use of single doses in uncomplicated influenza. It has demonstrated clinical benefit and potent antiviral activity in otherwise healthy and at-risk outpatients with acute influenza A and B. Whether this rapid antiviral efficacy might decrease virus transmission to contacts requires study. Its therapeutic use is associated with relatively high frequencies of emergence of variants with PA substitutions conferring reduced susceptibility. Baloxavir studies in children and in combination with SOC NAIs in hospitalized patients are underway. Combinations of an NAI and a polymerase inhibitor, or perhaps two polymerase inhibitors, offer promise for further enhancing antiviral potency, reducing resistance emergence, and potentially extending the treatment window and improving outcomes in key target populations.

## Acknowledgements

The authors thank Dr Carol Epstein, formerly of Medivector; Dr Lorant Leopold, Janssen; and Dr Takeki Uehara, Shionogi for their review of the manuscript and providing helpful comments and Lisa Cook, University of Virginia School of Medicine, for her help in manuscript preparation.

### Financial support and sponsorship

No direct funding was provided for this work. However, the review is based in part on an unpublished review of investigational influenza therapeutics that was commissioned and funded by WHO and written by F.G.H.

### Conflicts of interest

F.G.H. reports personal fees from WHO and from University of Alabama Antiviral Drug Discovery and Development Consortium: payments to the University of Virginia for his service on DSMBs for Celltrion, GSK, and Vaccitech; charitable donations from Shionogi, PrEP Biopharm, Cidara, and Seqirus for his consulting; travel support from Shionogi; and noncompensated consulting for various companies engaged in developing influenza therapeutics or vaccines (CoCrystal, Farmak, Genentech/Roche, GSK, Janssen, MedImmune, Medivector/FujiFilm, Regeneron, resTORbio, SAB Biotherapeutics, Vir, Visterra).

N.S. is a staff member of the WHO. She is responsible for the views expressed in this article and they do not necessarily represent the decisions, policy, or views of the WHO.

There are no conflicts of interest.

## REFERENCES AND RECOMMENDED READING

Papers of particular interest, published within the annual period of review, have been highlighted as:

▪ of special interest
▪▪ of outstanding interest
